# Molecular Epidemiology of Western Equine Encephalitis Virus, South America, 2023–2024

**DOI:** 10.3201/eid3009.240530

**Published:** 2024-09

**Authors:** Aline Scarpellini Campos, Ana Claúdia Franco, Fernanda M. Godinho, Rosana Huff, Darlan S. Candido, Jader da Cruz Cardoso, Xinyi Hua, Ingra M. Claro, Paola Morais, Carolina Franceschina, Thales de Lima Bermann, Franciellen Machado dos Santos, Milena Bauermann, Tainá Machado Selayaran, Amanda Pellenz Ruivo, Cristiane Santin, Juciane Bonella, Carla Rodenbusch, José Carlos Ferreira, Scott C. Weaver, Vilar Ricardo Gewehr, Gabriel Luz Wallau, William M. de Souza, Richard Steiner Salvato

**Affiliations:** Secretaria de Saúde do Estado do Rio Grande do Sul, Porto Alegre, Brazil (A.S. Campos, F.M. Godinho, R. Huff, J. da Cruz Cardoso, P. Morais, C. Franceschina, F. Machado dos Santos, M. Bauermann, T.M. Selayaran, A.P. Ruivo, R.S. Salvato);; Universidade Federal do Rio Grande do Sul, Porto Alegre (A.C. Franco, T. de L. Bermann, R.S. Salvato);; Imperial College London, London, UK (D.S. Candido);; University of Kentucky, Lexington, Kentucky, USA (X. Hua, I.M. Claro, W.M. de Souza);; Secretaria de Agricultura do Estado do Rio Grande do Sul, Porto Alegre (C. Santin, J. Bonella, C. Rodenbusch, J.C. Ferreira, V.R. Gewehr);; University of Texas Medical Branch, Galveston, Texas, USA (S.C. Weaver);; Fundação Oswaldo Cruz, Recife, Brazil (G.L. Wallau);; Bernhard Nocht Institute for Tropical Medicine, Hamburg, Germany (G.L. Wallau)

**Keywords:** Western equine encephalitis virus, viruses, mosquito-borne alphavirus, central nervous system infection, alphavirus, arbovirus, meningitis/encephalitis, alphavirus, vector-borne infections, South America, Argentina, Uruguay, Brazil, zoonoses

## Abstract

Western equine encephalitis virus (WEEV) is a mosquitoborne virus that reemerged in December 2023 in Argentina and Uruguay, causing a major outbreak. We investigated the outbreak using epidemiologic, entomological, and genomic analyses, focusing on WEEV circulation near the Argentina‒Uruguay border in Rio Grande do Sul state, Brazil. During November 2023‒April 2024, the outbreak in Argentina and Uruguay resulted in 217 human cases, 12 of which were fatal, and 2,548 equine cases. We determined cases on the basis of laboratory and clinical epidemiologic criteria. We characterized 3 fatal equine cases caused by a novel WEEV lineage identified through a nearly complete coding sequence analysis, which we propose as lineage C. Our findings highlight the importance of continued surveillance and equine vaccination to control future WEEV outbreaks in South America.

Western equine encephalitis virus (WEEV) is a mosquitoborne alphavirus that causes central nervous system (CNS) infection in humans and equids in the United States, Canada, and the southern cone of South America (*1*). WEEV is transmitted in enzootic and epizootic transmission cycles mainly by *Culex* and *Aedes* mosquitoes among birds and lagomorphs, which can lead to sporadic spillover to equids and humans (*2*,*3*). In humans, western equine encephalitis (WEE) infections are usually mild or asymptomatic, causing fever, headache, and myalgia (*4*). However, some patients experience encephalitis, which can be fatal in 5%–15% of cases (*5*). In equids, WEEV infection can cause neurologic disease (blindness, staggering, and seizures) with high case-fatality rates, often leading to death within days. As of July 2024, no specific treatments or vaccines are available to treat or prevent WEEV infection in humans; inactivated vaccines effectively prevent the disease in equids (*6*).

The largest WEEV outbreaks, which caused tens of thousands of equine and >3,000 human cases, were reported in the 1930s‒1940s. However, <700 confirmed cases were reported in the United States after the 1960s, and none has been reported during the past 25 years (*1*,*4*). Similarly, major outbreaks occurred in South America during the 1970s and 1980s, followed by isolated cases in Argentina in 1996 and Uruguay in 2009 (*1*,*7*,*8*). In December 2023, a large WEEV reemergence began with a major outbreak in Argentina and Uruguay. In this study, we contextualize the WEEV outbreak in Argentina and Uruguay and investigate active WEEV circulation in Rio Grande do Sul, Brazil, a state bordering those countries.

## Materials and Methods

### Epidemiologic Data

We obtained epidemiologic data of WEEV cases in equids and humans in Argentina and Uruguay from the Pan American Health Organization (*9*). The dataset included demographic and clinical characteristics and the aggregate number of human WEE cases in Argentina per epidemiologic week from epidemiologic week 43 (October 22‒28) in 2023 to epidemiologic week 23 (June 2‒8) in 2024. The equine WEE cases included laboratory-confirmed and suspected cases based on clinical or epidemiologic criteria.

### Equine Samples

We performed molecular screening in brain tissue samples from fatal horse cases, which were submitted to the Center for Health Surveillance of Rio Grande do Sul State during January 1, 2023‒April 10, 2024. All samples were stored at −80°C until testing (Appendix Table 1). 

### Entomologic Surveillance

We carried out entomologic surveillance in the Uruguaiana municipality in Rio Grande do Sul, focusing on 2 nearby horse breeding farms with a recent history of neurologic equine disease; we later confirmed 1 WEEV-positive case. We deployed 8 CDC light traps (John W. Hock Co., https://www.johnwhock.com) and 4 Biogents BG-Pro traps (Biogents, https://biogents.com) positioned at a height of 1.5 m above ground level, placed near horses and vegetation. We conducted mosquito sampling during 2 time periods at each site: a 24-hour period starting at 6 P.M. and a 12-hour period of 6 P.M.‒6 A.M. We flash-froze captured mosquitoes, stored them in liquid nitrogen, and transported for storage at −80°C at the Rio Grande do Sul State Center for Health Surveillance in Porto Alegre. We identified mosquito species morphologically using standard keys (*10*,*11*). We pooled mosquitoes by species and date, <10 mosquitoes per pool. We amplified the cytochrome oxidase I gene by PCR and sequenced for the molecular identification of mosquito pools, following previously described methods (*12*) (Appendix Table 2). 

### PCR Testing for Viruses

We immersed brain tissue fragments (≈2 g/cm^3^) in 800 µL of TRIzol reagent (ThermoFisher Scientific, https://www.thermofisher.com) and subjected to disruption using a Precellys 24 Touch (Thomas Scientific, https://www.thomassci.com). We immediately centrifuged the mixture to isolate the supernatant, from which we extracted viral RNA using the Extracta Kit Fast–DNA and RNA Viral (Loccus, https://www.loccus.com.br) according to the manufacturer’s instructions. We homogenized the mosquito pool samples with 800 µL of phosphate-buffered saline and extracted RNA using Extracta Kit Fast–DNA and RNA Viral. We tested all extracted RNA by quantitative real-time reverse transcription PCR (rRT-PCR) targeting WEEV using the TaqMan RNA to-CT 1-Step Kit (ThermoFisher Scientific), as previously described (*13*). We performed reactions on a CFX Opus 96 Real-Time PCR System (Bio-Rad Laboratories, https://www.bio-rad.com). In addition, we screened the samples using rRT-PCR targeting eastern equine encephalitis (*13*), West Nile, St. Louis encephalitis (*14*), Mayaro, and Oropouche viruses (*15*) (Appendix Table 3). We also tested equine brain tissue samples for rabies viruses (*16*).

### WEEV Genome Sequencing and Assembly

We conducted WEEV genome sequencing on 3 horse brain samples that tested positive by rRT-PCR. We achieved a near-complete genome using the hybrid–capture-based metagenomic approach enabled by Illumina Viral Surveillance Panel and RNA Prep with Enrichment kit (Illumina, https://www.illumina.com), according to the manufacturer's instructions. We sequenced VSP-enriched libraries on an Illumina MiSeq platform and processed the generated raw FASTQ files through the ViralFlow 1.0 pipeline (*17*) for assembly, using the 1971 Oregon WEEV strain 71V-1658 (GenBank accession no. NC_003908.1), as a reference genome.

### Phylogenetic Analysis

We generated 3 novel WEEV genomes with >98% coverage and aligned them with WEEV strains with complete coding sequences that were available in the GenBank database as of June 10, 2024. We performed multiple sequence alignment (MSA) using MAFFT version 7.450 (https://mafft.cbrc.jp/alignment/software) as previously described (*18*) and conducted manual adjustment using Geneious Prime 2023.0.4 (https://www.geneious.com). We screened the dataset for recombination events using all available methods in RDP version 4 (https://rdp4.software.informer.com) (*19*). We generated a maximum-likelihood (ML) phylogeny tree using IQ-TREE version 2 (http://www.iqtree.org) under a general time-reversible plus invariable plus gamma model determined by ModelFinder (*20*,*21*). We used the ultrafast-bootstrap approach with 1,000 replicates to determine the statistical support for nodes in the ML phylogeny (*22*). We estimated regressed root-to-tip genetic divergence against sampling dates to examine the temporal signal and identify sequences with low data quality of our datasets, such as assembly errors, sample contamination, data annotation errors, sequencing, and alignment errors (*23*). We identified no obvious outliers. We estimated the dated phylogenetic tree using BEAST version 1.10.4 (http://beast.community) under a general time-reversible plus invariable plus gamma model (*24*), an uncorrelated log-normal relaxed molecular clock (UCLD) model with an exponential rate distribution as previously described (*1*), and a Skygrid tree prior (*25*) with 102 grids with one grid every 2 years since the root of the tree. We used BEAGLE (http://beagle-lib.googlecode.com) to enhance computation speed (*26*). Last, we ran the evolutionary analyses independently in triplicate for 500 million steps, sampling parameters and trees every 50,000 steps. We generated maximum clade credibility summary trees using TreeAnnotator version 1.10.69 (https://beast.community/treeannotator) and visualized the phylogenetic tree by using Figtree version 1.4.2 (http://tree.bio.ed.ac.uk/software/figtree).

## Results

**Figure 1:**
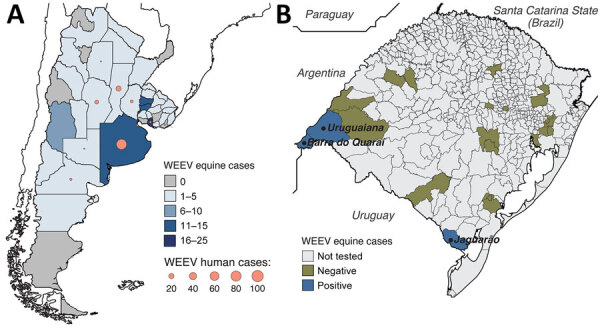
Western equine encephalitis cases in Argentina, Uruguay, and Brazil. A) Cumulative western equine encephalitis laboratory-confirmed cases in Argentina and Uruguay reported to the Pan American Health Organization (PAHO) during October 2023‒June 2024 (*9*). B) Locations of deaths among horses in Rio Grande do Sul state, Brazil, that tested positive (blue) and negative (yellow) for WEEV by RT-PCR during December 2023‒April 2024. The cases were identified by our molecular epidemiology study in Barra do Quaraí on December 21, 2023 (EQ1090), in Uruguaiana on December 28, 2023 (EQ1122), and in Jaguarão on January 30, 2024 (EQ237). WEEV, western equine encephalitis virus.

During November 18, 2023‒April 6, 2024, a major WEE outbreak occurred in Argentina and Uruguay, causing 112 human and 127 equine cases, all laboratory-confirmed (Figure 1, panel A). On the basis of clinical and epidemiologic criteria, Argentina also reported 68 suspected human cases and 1,481 suspected equine cases, and Uruguay documented 37 suspected human cases and 940 suspected equine cases. The WEE outbreak began in northeastern Argentina, spreading to central regions and Uruguay. In Argentina, 47 confirmed equine cases were reported across 17 of 23 provinces, peaking in epidemiologic week 49 of 2023 and declining sharply by epidemiologic week 8 of 2024. A total of 107 human WEE cases, including 12 fatal cases, were identified in 8 provinces of Argentina; 63/107 (58.9%) were concentrated in Buenos Aires Province, mirroring the highest equine case burden of 14/47 (29.8%) (Figure 1, panel A). The human outbreak peaked between epidemiologic week 51 of 2023 and epidemiologic week 3 of 2024; during that period, 58/107 (54.2%) of cases occurred. Available data show that the human WEE case-patients were predominantly male (male-to-female ratio 6.6:1), and 76/106 (71.7%) were >50 years of age. The most common symptoms were fever (82%), headache (76%), and mental confusion (63%). Uruguay exhibited a similar pattern; the equine outbreak preceded human cases. The largest proportion of confirmed equine cases (28.8%, 23/80) occurred in San José Department, which also reported 3/5 confirmed human cases (Figure 1, panel A). No WEEV cases were reported in Argentina and Uruguay between epidemiologic week 15 of 2023 (April 7‒14) and epidemiologic week 24 of 2024 (June 11‒17).

During December 2023‒April 2024, we conducted a molecular diagnostic and entomologic study to investigate the presence of WEEV in Rio Grande do Sul state, Brazil. In the entomologic surveillance, we captured 971 mosquitoes across 7 genera that were combined into 117 pools for further analysis (Appendix Table 2). The most prevalent genus was *Culex*, constituting 664/971 (68.4%) mosquitoes. All mosquito pools tested negative for WEEV, eastern equine encephalitis, West Nile, St. Louis encephalitis, Mayaro, and Oropouche viruses.

During January 1, 2023–April 10, 2024, we received brain samples from 31 fatal horse cases from 22/497 (4.4%) municipalities in Rio Grande do Sul. We tested 31 horse brain tissue samples by rRT-PCR and detected WEEV RNA in 3 (9.7%) with cycle threshold (Ct) values of 26–27 (Figure 1, panel B; Appendix Table 1). All 3 horses exhibited signs of neurologic disease (i.e., paralysis and incoordination) and none was vaccinated against WEEV. One horse was 2 months of age and died on December 21, 2023, in Barra do Quaraí municipality; the second was 2 years of age and died December 28 in Uruguaiana municipality. Those municipalities are located on the Brazil border with Argentina and Uruguay. The third case was a 5-month-old horse that died on January 30, 2024, in Jaguarão municipality, on the Brazil–Uruguay border. In addition, those 3 fatal horse cases were positive for rabies virus by rRT-PCR; the etiologic cause of death remained inconclusive for the other 22 fatal horse cases (Appendix Table 1).

**Figure 2:**
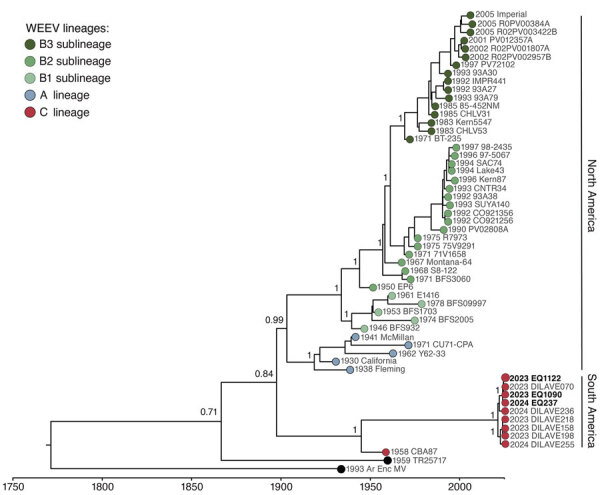
Maximum-likelihood phylogenetic tree of 3 new WEEV strains from Rio Grande do Sul state, Brazil (bold text), and reference sequences. Tip colors indicate WEEV lineage. We used an uncorrelated log-normal relaxed molecular clock model with an exponential rate distribution for generating the time-rooted tree. Posterior probability scores appear next to key well-supported nodes. Dates at key nodes are the estimated dates of divergence from a common ancestor, with Bayesian credible intervals. WEEV, western equine encephalitis virus.

**Figure 3:**
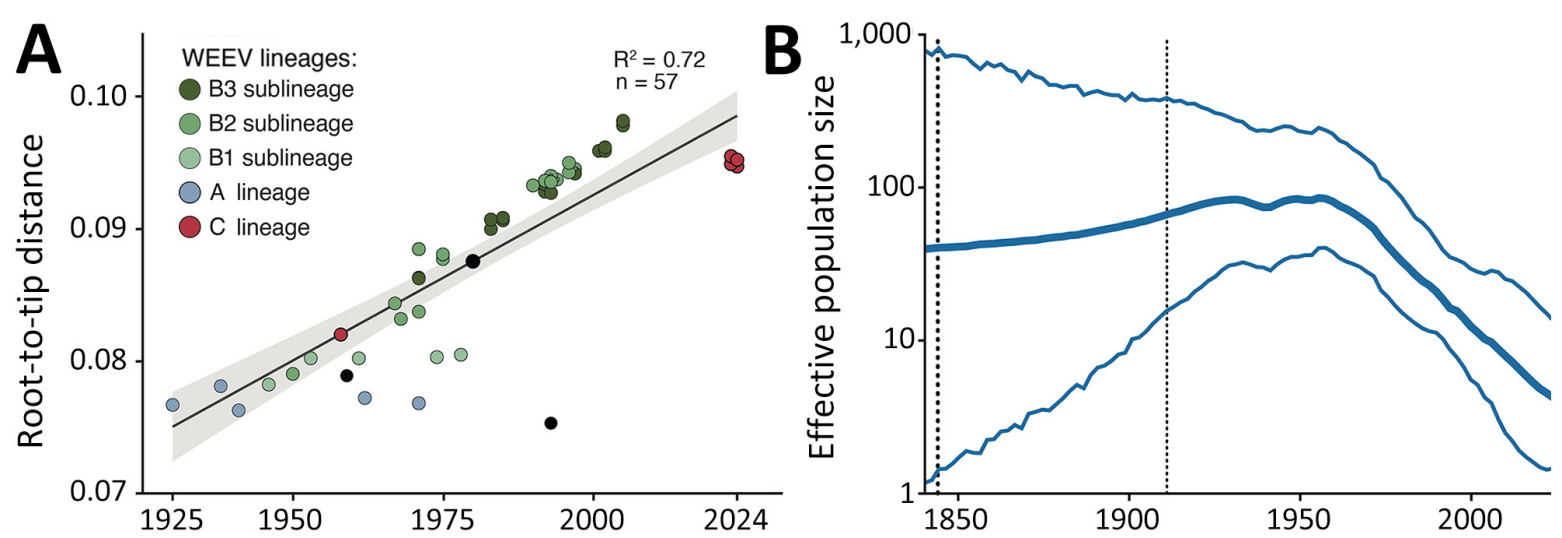
Phylogenetic analysis of WEEV strains from Rio Grande do Sul state, Brazil, and reference sequences. A) Regression of sequence sampling dates against root-to-tip genetic distances in a maximum likelihood phylogeny of the WEEV strains. Sequences are colored according to geographic source. Line indicates the correlation; shading indicates 95% CIs. B) Effective population size of WEEV through time using the Skygrid model. Thin blue lines represent 95% Bayesian credibility interval, and the thick blue line represents the posterior median. Vertical dotted lines indicate the best estimates for the time of the root of the tree (left) and the upper highest posterior density (right). WEEV, western equine encephalitis virus.

Next, we used Illumina sequencing to generate the nearly complete (>98%) coding sequences for 3 WEEV strains with a mean depth of coverage of 606-fold/nt. We submitted sequences to GenBank (accession nos. PP544260, PP669617, and PP66961). The maximum-likelihood phylogenetic analysis showed that 3 WEEV strains circulating in the Rio Grande do Sul state in 2024 clustered together in a well-supported clade (posterior probability = 1) with 6 genome sequences of WEEV obtained from equine cases in Uruguay during the outbreak of 2023–2024 (Figure 2). That clade represents a novel WEEV lineage closely related to the 1958 CBA87 strain from Argentina, which we proposed as the C lineage (Figure 2). We confirmed that our genomic dataset had a strong temporal signal based on regression of genetic divergence from root-to-tip against sample collection dates (R_2_ = 0.7763) (Figure 3, panel A). Those novel WEEV strains from Brazil shared 94.9‒97.6% nucleotide identity with the 1958 CBA87 strain (Appendix Table 2). The time to the most recent common ancestor (tMRCA) for WEEV strains associated with the 2023–2024 outbreak in Brazil was estimated to be early 2019 (95% Bayesian credible interval early 2012 to mid-2022) with a mean evolutionary rate of 2.6 × 10^−4^ substitutions/nucleotide/year (UCLD, mean 2.6 × 10^−4^, median: 2.6 × 10^−4^; 95% height posterior density 1.8 × 10^−4^ – 3.5 × 10^−4^ substitutions/nucleotide/year). Using Skygrid reconstruction with a grid with an interval of 2 years based on tMRCA for all WEEV, we found an effective population size that has been decreasing between the late 1960s and 1980s (Figure 3, panel B). We found no evidence of recombination in WEEV strains of the C lineage.

## Discussion

We contextualized the major WEE outbreak in South America in 2023‒2024 and identified a new WEEV lineage associated with this outbreak from 3 fatal horse cases from Rio Grande do Sul, Brazil. The new lineage forms a distinct clade evolving independently for many decades from those circulating in North America (*1*,*4*,*27*). Our findings strongly suggest that WEEV has been circulating in South America since 2009 with cases likely unreported. We hypothesize 2 complementary explanations for the lack of reported WEE cases from April 2009‒November 2023 in South America: limited enzootic circulation of the WEEV between vectors and resident/migratory birds, or a lack of WEEV surveillance, particularly in rural or remote areas, where cases might go undetected. The WEE outbreak in South America appears to have ended in April 2024, when the last case was reported. This cessation of cases may be due to the increased or mandatory immunization of horses against WEE in Argentina, Uruguay, and Brazil. Widespread equine immunization in the region may contribute to suppressing WEEV re-emergence in the near future.

Some experimental murine model studies indicate reduced virulence of recent WEEV isolates (B3 strains) compared with earlier isolates (A, B1, B2 strains), which could explain decreased human and equine cases in North America (*1*,*4*,*28*). Alternatively, the lack of reported WEE cases in North America since 1999 may a result of WEEV’s silent or underreported circulation, because WEEV continued to be detected in mosquitoes during 2004–2007 (*29*). In contrast, the large numbers of neurologic and fatal cases in humans and equids in Argentina, Brazil, and Uruguay in 2023–2024 suggest that contemporary WEEV strains circulating in South America might be more virulent than those currently circulating in North America. Further research is needed to elucidate the virulence determinants that might explain this apparent difference between WEEV lineages from South and North America.

The first limitation of our study is that we focused on identifying active WEEV infections using molecular methods only in fatal horse cases. Horses with mild signs of disease should also be tested. Also, age-stratified serologic studies are needed to determine the extent of previous WEEV exposure in the equine and human populations, and patterns of past infections. Second, we did not detect WEEV RNA in any mosquitoes, which may be explained by the generally low (e.g., <1%) arbovirus infection rates observed in mosquitoes, even during outbreaks, and including WEEV. For instance, 0.02% (55/271,889) of mosquitoes captured in California, USA, during 2004–2013 were positive for WEEV by rRT-PCR (*29*). Also, our entomologic investigation showed that *Culex* spp. were the most abundant mosquito species in the region; further studies with an emphasis on *Culex* spp. mosquitoes, as well as the *Aedes albifasciatus* mosquito, a previously incriminated WEEV vector (*3*,*30*), are needed to better understand the transmission dynamics of WEEV in South America. Third, we were unable to determine whether ecologic drivers were associated with the current outbreaks, but further studies should investigate climate factors, anthropogenic changes, and migratory bird routes and activity (*31*).

In conclusion, our study identified active WEEV circulation in Rio Grande do Sul, Brazil, and a novel viral lineage associated with fatal cases in horses. These findings highlight the critical need for continuous laboratory diagnosis and surveillance for WEEV in both horses and humans, as well as ecologic studies using a One Health approach to better understand the transmission dynamics and ecologic drivers contributing to WEEV re-emergence in South America. Finally, horse immunization should be considered to mitigate the effect on animal health.

This article was preprinted at https://www.medrxiv.org/content/10.1101/2024.04.15.24305848v1.

Appendix Additional information about molecular epidemiology of western equine encephalitis virus, South America, 2023–2024.

## References

[1] Bergren NA, Auguste AJ, Forrester NL, Negi SS, Braun WA, Weaver SC. Western equine encephalitis virus: evolutionary analysis of a declining alphavirus based on complete genome sequences. J Virol. 2014;88:9260–7. 10.1128/JVI.01463-14 24899192 PMC4136285

[2] Calisher CH, Monath TP, Mitchell CJ, Sabattini MS, Cropp CB, Kerschner J, et al. Arbovirus investigations in Argentina, 1977-1980. III. Identification and characterization of viruses isolated, including new subtypes of western and Venezuelan equine encephalitis viruses and four new bunyaviruses (Las Maloyas, Resistencia, Barranqueras, and Antequera). Am J Trop Med Hyg. 1985;34:956–65. 10.4269/ajtmh.1985.34.956 2863990

[3] Avilés G, Sabattini MS, Mitchell CJ. Transmission of western equine encephalomyelitis virus by Argentine *Aedes albifasciatus* (Diptera: Culicidae). J Med Entomol. 1992;29:850–3. 10.1093/jmedent/29.5.850 1404265

[4] Bergren NA, Haller S, Rossi SL, Seymour RL, Huang J, Miller AL, et al. “Submergence” of Western equine encephalitis virus: Evidence of positive selection argues against genetic drift and fitness reductions. PLoS Pathog. 2020;16:e1008102. 10.1371/journal.ppat.1008102 32027727 PMC7029877

[5] Cohen R, O’Connor RE, Townsend TE, Webb PA, McKey RW. Western equine encephalomyelitis; clinical observations in infants and children. J Pediatr. 1953;43:26–34. 10.1016/S0022-3476(53)80084-3 13062069

[6] Bartelloni PJ, McKinney RW, Calia FM, Ramsburg HH, Cole FE Jr. Inactivated western equine encephalomyelitis vaccine propagated in chick embryo cell culture. Clinical and serological evaluation in man. Am J Trop Med Hyg. 1971;20:146–9. 10.4269/ajtmh.1971.20.146 5105896

[7] Avilés G, Bianchi TI, Daffner JF, Sabattini MS. [Post-epizootic activity of Western equine encephalitis virus in Argentina] [in Spanish]. Rev Argent Microbiol. 1993;25:88–99. 8234736

[8] Delfraro A, Burgueño A, Morel N, González G, García A, Morelli J, et al. Fatal human case of Western equine encephalitis, Uruguay. Emerg Infect Dis. 2011;17:952–4. 10.3201/eid1705.101068 21529429 PMC3321764

[9] Pan American Health Organization. Western equine encephalitis in the Region of the Americas [in Spanish]. 2024 [cited 27 Jul 2024]. https://shiny.paho-phe.org/encephalitis

[10] Consoli RAGB, Lourenço de Oliveira R. Main mosquitoes of health importance in Brazil [in Portuguese]. 2nd edition. Rio de Janeiro: FIOCRUZ; 1998.

[11] Forattini OP. Medical culicidology. Vol 2. Identification, biology, epidemiology. Sao Paulo: University of Sao Paulo; 2002.

[12] Muñoz-Gamba AS, Laiton-Donato K, Perdomo-Balaguera E, Castro LR, Usme-Ciro JA, Parra-Henao G. Molecular characterization of mosquitoes (Diptera: Culicidae) from the Colombian rainforest. Rev Inst Med Trop São Paulo. 2021;63:e24. 10.1590/s1678-9946202163024 33787744 PMC7997665

[13] Lambert AJ, Martin DA, Lanciotti RS. Detection of North American eastern and western equine encephalitis viruses by nucleic acid amplification assays. J Clin Microbiol. 2003;41:379–85. 10.1128/JCM.41.1.379-385.2003 12517876 PMC149608

[14] Lanciotti RS, Kerst AJ. Nucleic acid sequence-based amplification assays for rapid detection of West Nile and St. Louis encephalitis viruses. J Clin Microbiol. 2001;39:4506–13. 10.1128/JCM.39.12.4506-4513.2001 11724870 PMC88574

[15] Naveca FG, Nascimento VAD, Souza VC, Nunes BTD, Rodrigues DSG, Vasconcelos PFDC. Multiplexed reverse transcription real-time polymerase chain reaction for simultaneous detection of Mayaro, Oropouche, and Oropouche-like viruses. Mem Inst Oswaldo Cruz. 2017;112:510–3. 10.1590/0074-02760160062 28591313 PMC5452489

[16] Wadhwa A, Wilkins K, Gao J, Condori Condori RE, Gigante CM, Zhao H, et al. A pan-lyssavirus Taqman real-time RT-PCR assay for the detection of highly variable rabies virus and other lyssaviruses. PLoS Negl Trop Dis. 2017;11:e0005258. 10.1371/journal.pntd.0005258 28081126 PMC5230753

[17] Dezordi FZ, Neto AMDS, Campos TL, Jeronimo PMC, Aksenen CF, Almeida SP, et al.; On Behalf Of The Fiocruz Covid-Genomic Surveillance Network. ViralFlow: a versatile automated workflow for SARS-CoV-2 genome assembly, lineage assignment, mutations and intrahost variant detection. Viruses. 2022;14:217. 10.3390/v14020217 35215811 PMC8877152

[18] Katoh K, Standley DM. MAFFT multiple sequence alignment software version 7: improvements in performance and usability. Mol Biol Evol. 2013;30:772–80. 10.1093/molbev/mst010 23329690 PMC3603318

[19] Martin DP, Murrell B, Golden M, Khoosal A, Muhire B. RDP4: Detection and analysis of recombination patterns in virus genomes. Virus Evol. 2015;1:vev003. 10.1093/ve/vev003 27774277 PMC5014473

[20] Minh BQ, Schmidt HA, Chernomor O, Schrempf D, Woodhams MD, von Haeseler A, et al. IQ-TREE 2: new models and efficient methods for phylogenetic inference in the genomic era. Mol Biol Evol. 2020;37:1530–4. 10.1093/molbev/msaa015 32011700 PMC7182206

[21] Kalyaanamoorthy S, Minh BQ, Wong TKF, von Haeseler A, Jermiin LS. ModelFinder: fast model selection for accurate phylogenetic estimates. Nat Methods. 2017;14:587–9. 10.1038/nmeth.4285 28481363 PMC5453245

[22] Hoang DT, Chernomor O, von Haeseler A, Minh BQ, Vinh LS. UFBoot2: improving the ultrafast bootstrap approximation. Mol Biol Evol. 2018;35:518–22. 10.1093/molbev/msx281 29077904 PMC5850222

[23] Rambaut A, Lam TT, Max Carvalho L, Pybus OG. Exploring the temporal structure of heterochronous sequences using TempEst (formerly Path-O-Gen). Virus Evol. 2016;2:vew007. 10.1093/ve/vew007 27774300 PMC4989882

[24] Suchard MA, Lemey P, Baele G, Ayres DL, Drummond AJ, Rambaut A. Bayesian phylogenetic and phylodynamic data integration using BEAST 1.10. Virus Evol. 2018;4:vey016. 10.1093/ve/vey016 29942656 PMC6007674

[25] Gill MS, Lemey P, Faria NR, Rambaut A, Shapiro B, Suchard MA. Improving Bayesian population dynamics inference: a coalescent-based model for multiple loci. Mol Biol Evol. 2013;30:713–24. 10.1093/molbev/mss265 23180580 PMC3563973

[26] Ayres DL, Darling A, Zwickl DJ, Beerli P, Holder MT, Lewis PO, et al. BEAGLE: an application programming interface and high-performance computing library for statistical phylogenetics. Syst Biol. 2012;61:170–3. 10.1093/sysbio/syr100 21963610 PMC3243739

[27] Weaver SC, Kang W, Shirako Y, Rumenapf T, Strauss EG, Strauss JH. Recombinational history and molecular evolution of western equine encephalomyelitis complex alphaviruses. J Virol. 1997;71:613–23. 10.1128/jvi.71.1.613-623.1997 8985391 PMC191092

[28] Mossel EC, Ledermann JP, Phillips AT, Borland EM, Powers AM, Olson KE. Molecular determinants of mouse neurovirulence and mosquito infection for Western equine encephalitis virus. PLoS One. 2013;8:e60427. 10.1371/journal.pone.0060427 23544138 PMC3609757

[29] Brault AC, Fang Y, Reisen WK. Multiplex qRT-PCR for the detection of western equine encephalomyelitis, St. Louis encephalitis, and West Nile viral RNA in mosquito pools (Diptera: Culicidae). J Med Entomol. 2015;52:491–9. 10.1093/jme/tjv021 26334826 PMC4581483

[30] Cardoso J, Corseuil E, Barata JMS. Culicinae (Diptera, Culicidae) occurring in the state of Rio Grande do Sul, Brasil [in Portuguese]. Rev Bras Entomol. 2005;49:275–87. 10.1590/S0085-56262005000200013

[31] de Souza WM, Weaver SC. Effects of climate change and human activities on vector-borne diseases. Nat Rev Microbiol. 2024;22:476–91. 10.1038/s41579-024-01026-0 38486116

