# Supplementary material for: Molecular Epidemiology of Western Equine Encephalitis Virus, South America, 2023–2024
Source: Emerg Infect Dis. 2024 Sep;30(9):1834–40. doi: 10.3201/eid3009.240530 (PMC11346983; doi:10.3201/eid3009.240530)
Supplement: Appendix — Additional information about molecular epidemiology of western equine encephalitis virus, South America, 2023–2024. [file 24-0530-Techapp-s1.pdf]

Article DOI: http://doi.org/10.3201/eid3009.240530

EID cannot ensure accessibility for supplementary materials supplied by authors. Readers who have difficulty accessing supplementary content should contact the authors for assistance.

Molecular Epidemiology of Western Equine Encephalitis Virus, South America, 2023–2024

Appendix

Appendix Table 1. Characteristics of samples of horses exhibiting neurological signs submitted to the Rio Grande do Sul State Center for Health Surveillance*

Municipality	Date of collection	Age	WEEV	Rabies Ct-value
Morro Redondo	24-Jan-2023	11 years	ND	ND
Pelotas	24-Jan-2023	15 years	ND	ND
Gravataí	17-Feb-2023	14 years	ND	ND
Taquara	4-Apr-2023	4 years	ND	ND
Parobé	10-Apr-2023	2 years	ND	ND
Pantano Grande	2-May-2023	8 years	ND	ND
Rio Pardo	10-May-2023	12 years	ND	Positive
Alegrete	10-May-2023	5 years	ND	ND
Porto Alegre	30-May-2023	N/A	ND	ND
Porto Alegre	30-May-2023	N/A	ND	ND
Igrejinha	20-June-2023	4 years	ND	ND
Porto Alegre	21-June-2023	11 years	ND	ND
Porto Alegre	13-July-2023	12 years	ND	ND
Glorinha	28-July-2023	N/A	ND	ND
Porto Alegre	22-Aug-2023	23 years	ND	ND
Bossoroca	24-Aug-2023	5 years	ND	ND
Marau	19-Sep-2023	8 years	ND	ND
Boqueirão do Leão	28-Nov-2023	N/A	ND	ND
Boqueirão do Leão	28-Nov-2023	N/A	ND	ND
Arroio do Meio	13-Dec-2023	12 years	ND	ND
Barra do Quaraí	21-Dec-2023	2 months	Positive (Ct-value 26)	ND
São Miguel das Missões	27-Dec-2023	4 years	ND	Positive
Caxias do Sul	27-Dec-2023	14 years	ND	ND
Bagé	28-Dec-2023	2 months	ND	ND
Uruguaiana	28-Dec-2023	2 years	Positive (Ct-value 27)	ND
Alegrete	5-Jan-2024	N/A	ND	Positive
Jaguarão	30-Jan-2024	5 months	Positive (Ct-value 27)	ND
Porto Alegre	5-Feb-2024	N/A	ND	ND
Porto Alegre	16-Feb-2024	12 years	ND	ND
Itaqui	21-Feb-2024	14 years	ND	ND
Gravataí	6-Mar-2024	N/A	ND	ND

*Ct-value, cycle threshold value; N/A, unavailable; ND, Not detected; WEEV, Western equine encephalitis virus.

Appendix Table 2. Characteristics of mosquitoes collecting during the entomological surveillance in Uruguaiana municipality, Rio Grande do Sul State, Brazil

ID	Pool size	Species
MQ1.1	1	*Aedeomyia squamipennis*
MQ1.2	1	*Mansonia sp.*
MQ1.3	10	*Culex sp.*
MQ1.4	4	*Culex sp.*
MQ10	1	*Culex sp.*
MQ11	2	*Culex pipiens*
MQ12.1	1	*Culex coronator*
MQ12.2	1	*Culex maxi*
MQ2.1	10	*Culex pipiens*
MQ2.10	10	*Culex pipiens*
MQ2.11	10	*Anopheles deaneorum*
MQ2.12	10	*Anopheles albitarsis*
MQ2.13	10	*Mansonia titillans*
MQ2.14	10	*Mansonia titillans*
MQ2.15	10	*Culex coronator*
MQ2.16	10	*Culex coronator*
MQ2.17	10	*Culex maxi*
MQ2.18	10	*Uranotaenia lowii*
MQ2.19	10	*Culex coronator*
MQ2.2	10	*Culex coronator*
MQ2.20	10	*Culex pipiens*
MQ2.21	10	*Mansonia titillans*
MQ2.22	10	*Culex coronator*
MQ2.23	10	*Culex coronator*
MQ2.24	10	*Mansonia titillans*
MQ2.25	10	*Anopheles deaneorum*
MQ2.26	10	*Culex coronator*
MQ2.27	10	*Culex coronator*
MQ2.28	10	*Culex coronator*
MQ2.29	10	*Culex interfor*
MQ2.3	10	*Culex pipiens*
MQ2.30	10	*Uranotaenia lowii*
MQ2.31	10	*Culex pipiens*
MQ2.32	10	*Culex pipiens*
MQ2.33	10	*Culex coronator*
MQ2.34	10	*Culex pipiens*
MQ2.35	10	*Culex pipiens*
MQ2.36	10	*Anopheles albitarsis*
MQ2.37	10	*Anopheles albitarsis*
MQ2.38	5	*Aedeomyia squamipennis*
MQ2.39	5	*Culex coronator*
MQ2.4	10	*Culex coronator*
MQ2.40	3	*Culex intrincatus*
MQ2.41	1	*Psorophora confinnis*
MQ2.42	2	*Aedes crinifer*
MQ2.43	1	*Culex coronator*
MQ2.5	10	*Culex coronator*
MQ2.6	10	*Mansonia sp.*
MQ2.7	10	*Culex pipiens*
MQ2.8	10	*Culex pipiens*
MQ2.9	10	*Culex sp.*
MQ3.1	10	*Culex pipiens*
MQ3.2	5	*Culex pipiens*
MQ4.1	10	*Culex coronator*
MQ4.10	10	*Culex coronator*
MQ4.11	10	*Culex pipiens*
MQ4.12	10	*Anopheles albitarsis*
MQ4.13	10	*Anopheles albitarsis*
MQ4.14	10	*Uranotaenia lowii*
MQ4.15	10	*Culex pipiens*
MQ4.16	10	*Culex pipiens*
MQ4.17	10	*Culex coronator*
MQ4.18	10	*Anopheles deaneorum*
MQ4.19	10	*Culex coronator*
MQ4.2	10	*Culex interfor*
MQ4.20	10	*Culex pipiens*
MQ4.21	10	*Uranotaenia lowii*

ID	Pool size	Species
MQ4.22	10	*Anopheles deaneorum*
MQ4.23	10	*Culex coronator*
MQ4.24	10	*Culex pipiens*
MQ4.25	10	*Uranotaenia lowii*
MQ4.26	10	*Anopheles albitarsis*
MQ4.27	10	*Culex coronator*
MQ4.28	10	*Culex coronator*
MQ4.29	10	*Culex coronator*
MQ4.3	10	*Culex sp.*
MQ4.30	10	*Anopheles deaneorum*
MQ4.31	10	*Culex coronator*
MQ4.32	6	*Culex maxi*
MQ4.33	4	*Psorophora confinnis*
MQ4.34	4	*Psorophora confinnis*
MQ4.4	10	*Anopheles albitarsis*
MQ4.5	10	*Uranotaenia lowii*
MQ4.6	10	*Culex pipiens*
MQ4.7	10	*Culex coronator*
MQ4.8	10	*Anopheles deaneorum*
MQ4.9	2	*Aedes scapularis*
MQ5	1	*Culex coronator*
MQ6	6	*Culex pipiens pallens*
MQ7.1	10	*Culex pipiens*
MQ7.10	10	*Culex pipiens*
MQ7.11	5	*Anopheles albitarsis*
MQ7.12	1	*Aedes scapularis*
MQ7.13	2	*Ochlerotatus scapularis*
MQ7.14	2	*Aedes aegypti*
MQ7.2	10	*Culex pipiens*
MQ7.3	10	*Culex pipiens*
MQ7.4	10	*Anopheles darlingi*
MQ7.5	10	*Anopheles sp.*
MQ7.6	6	*Aedes scapularis*
MQ7.7	10	*Culex pipiens*
MQ7.8	10	*Culex pipiens*
MQ7.9	4	*Culex pipiens*
MQ8.1	10	*Culex pipiens*
MQ8.10	10	*Culex pipiens*
MQ8.11	10	*Culex pipiens*
MQ8.12	10	*Culex pipiens*
MQ8.13	10	*Culex pipiens*
MQ8.14	7	*Culex bidens*
MQ8.2	10	*Culex pipiens*
MQ8.3	10	*Culex pipiens*
MQ8.4	7	*Culex pipiens*
MQ8.5	1	*Anopheles albitarsis*
MQ8.6	10	*Aedes albopictus*
MQ8.7	10	*Culex pipiens*
MQ8.8	10	*Culex pipiens*
MQ8.9	10	*Culex pipiens*

Appendix Table 3. Primers and probes were used for the detection of viruses in this study*

Virus	Sequences (5'→3')	Primers and probes	Target	Ref.
RABV	ACGCTTAACAACCAGATCAAAGAA	Forward	LN34	(Wadhwa et al., 2017) (*1*)
	ACGCTTAACAACAAAATCADAGAAG	Forward	LN34	
	CMGGGTAYTTRTAYTCATAYTGRTC	Reverse	N34	
	FAM-AACACCYCTACAATGGA-BHQ1	Probe	N34	
	FAM AACACTACTACAATGGA-BHQ1	Probe	N34	
	CGATGAAGATCAAGATCATTGC	Forward	β-Actin	
	AAGCATTTGCGGTGGAC	Reverse	β-Actin	
	HEX-TCCACCTTCCAGCAGATGTGGATCA-BHQ1	Probe	β-Actin	
MAYV	CACGGACMTTTTGCCTTCA	Forward	NSP1	Naveca et al., 2017 (*2*)
	AGACTGCCACCTCTGCTKGAG	Reverse	NSP1	
	VIC-ACAGATCAGACATGCAGG	Probe	NSP1	
OROV	TCCGGAGGCAGCATATGTG	Forward	NP	Naveca et al., 2017 (*2*)
	ACAACACCAGCATTGAGCACTT	Reverse	NP	
	FAM-CATTTGAAGCTAGATACGG	Probe	NP	
WNV	CAGACCACGCTACGGCG	Forward	E	Lanciotti et al., 2001 (*3*)
	CTAGGGCCGCGTGGG	Reverse	E	
	TCTGCGGAGAGTGCAGTCTGCGAT	Probe	E	
SLEV	CTGGCTGTCGGAGGGATTCT	Forward	E	Lanciotti et al., 2001 (*3*)
	TAGGTCAATTGCACATCCCG	Reverse	E	
	TCTGGCGACCAGCGTGCAAGCCG	Probe	E	
EEEV	ACACCGCACCCTGATTTTACA	Forward	E2	Lambert et al., 2001 (*4*)
	CTTCCAAGTGACCTGGTCGTC	Reverse	E2	
	TGCACCCGGACCATCCGACCT	Probe	E2	
WEEV	CTGAAAGTCGGCCTGCGTAT	Forward	E2	Lambert et al., 2001 (*4*)
	CGCCATTGACGAACGTATCC	Reverse	E2	
	ATACGGCAATACCACCGCGCACC	Probe	E2	

* MAYV, Mayaro virus; OROV, Oropouche virus; RABV, rabies virus; SLEV, St Louis encephalitis virus; WEEV, Western equine encephalitis virus; WNV, West Nile virus.

Appendix Table 4. Genome sequences used in the phylogenetic analysis

Accession	Isolate	Location	Host	Year	Lineage
GQ287640	McMillan	Canada: Ontario	Homo sapiens	1941	A
KJ554965	California	USA: San Joaquin Valley, CA	Equus caballus	1930	A
KT844544	Y62-33	Russia: Urdmurt	Aedes cinereus	1962	A
KT844545	CU71-CPA	Cuba		1971	A
MN477208	Fleming	USA	Homo sapiens	1938	A
GQ287644	BFS-2005	USA: Kern County, California	Culex tarsalis	1974	B1
KJ554966	BFS932	USA: Bakersfield, CA	Culex tarsalis	1946	B1
KJ554968	BFS1703	USA: Bakersfield, CA	Culex tarsalis	1953	B1
KJ554969	E1416	USA: Kern Co., CA	Zonotrichia leucophrys	1961	B1
KJ554974	BFS09997	USA: Kern Co., CA	Culex tarsalis	1978	B1
KJ554973	75V9291	USA: Wilkin City, MN	Culex tarsalis	1975	B2
KJ554977	PV02808A	USA: Lubbock Co., TX	Culicidae	1990	B2
KJ554979	CO921356	USA: Larimer City, CO	Culex tarsalis	1992	B2
KJ554980	93A38	USA: Tacna, AZ	Culicidae	1992	B2
KJ554984	CNTR34	USA: Contra Costa Co., CA	Culex tarsalis	1993	B2
KJ554985	Lake43	USA: Lake Co., CA	Culex tarsalis	1994	B2
KT844546	CO92-1256	USA: Colorado, Larimer City	Culex tarsalis	1992	B2
KT844548	SUYA140	USA: California, Sutter County	Culex tarsalis	1993	B2
KT844549	SAC74	USA: California, Sacramento County	Culex tarsalis	1994	B2
KT844550	Kern87	USA: California, Kern County	Culex tarsalis	1996	B2
KU978771	97-5067	USA	Meleagris gallopavo	1996	B2
KU978772	98-2435	USA	Dromaius novaehollandiae	1997	B2
NC_003908	71V1658	USA: Oregon		1971	B2
OQ184867	R7973	USA: Larimer County, Colorado	Homo sapiens	1975	B2

Accession	Isolate	Location	Host	Year	Lineage
KJ554967	EP6	USA: Missouri	Culicidae	1950	B2
GQ287641	Imperial	USA: Imperial County, California	Culex tarsalis	2005	B3
GQ287643	Montana-64	USA: Montana	Equus caballus	1967	B3
GQ287647	85-452NM	USA: New Mexico	Culex tarsalis	1985	B3
KJ554970	S8-122	USA: Butte Co., CA	Hesperosciurus griseus	1968	B3
KJ554971	BT-235	USA: Texas	Gopherus berlandieri	1971	B3
KJ554972	BFS3060	USA: Butte Co., CA	Culex tarsalis	1971	B3
KJ554975	KERN5547	USA: Kern Co., CA	Culex tarsalis	1983	B3
KJ554976	CHLV53	USA: Riverside Co., CA	Culex tarsalis	1983	B3
KJ554978	IMPR441	USA: Imperial Co., CA	Culex tarsalis	1992	B3
KJ554981	93A27	USA: Parker, AZ	Culicidae	1992	B3
KJ554982	93A30	USA: Phoenix, AZ	Culicidae	1993	B3
KJ554983	93A79	USA: Yuma, AZ	Culicidae	1993	B3
KJ554986	PV72102	USA: El Paso Co., TX	Culicidae	1997	B3
KJ554987	PV012357A	USA: El Paso Co., TX	Culicidae	2001	B3
KJ554988	R02PV002957B	USA: El Paso Co., TX	Culicidae	2002	B3
KJ554989	R02PV001807A	USA: El Paso Co., TX	Culicidae	2002	B3
KJ554990	R02PV003422B	USA: El Paso Co., TX	Culicidae	2005	B3
KJ554991	R0PV00384A	USA: El Paso Co., TX	Culicidae	2005	B3
KT844547	CHLV31	USA: California, Riverside County	Culex tarsalis	1985	B3
KT844543	CBA87	Argentina: Cordoba Pr., Oncativo	Equus ferus	1958	C
PP544260	EQ1090	Brazil: Rio Grande do Sul	Equus caballus	2023	C
PP669618	EQ1122	Brazil: Rio Grande do Sul	Equus caballus	2023	C
PP669617	EQ237	Brazil: Rio Grande do Sul	Equus caballus	2024	C
PP620641	DILAVE070	Uruguay: Paysandu	Equus caballus	2023	C
PP620642	DILAVE236	Uruguay: San Jose	Equus caballus	2023	C
PP620643	DILAVE218	Uruguay: San Jose	Equus caballus	2023	C
PP620644	DILAVE158	Uruguay: Paysandu	Equus caballus	2023	C
PP620645	DILAVE198	Uruguay: Rocha	Equus caballus	2024	C
PP620646	DILAVE255	Uruguay: San Jose	Equus caballus	2024	C
KT844541	TR25717	Guyana	Equus ferus	1959	none
KT844542	Ar_Enc_MV	Argentina: Buenos Aires Pr., Monte Veloz	Equus ferus	1993	none
NC_075015	AG80-646	Argentina: Chaco Province	Culex	1980	none

References

1. Wadhwa A, Wilkins K, Gao J, Condori Condori RE, Gigante CM, Zhao H, et al. A Pan-Lyssavirus Taqman Real-Time RT-PCR Assay for the Detection of Highly Variable Rabies virus and Other Lyssaviruses. PLoS Negl Trop Dis. 2017;11:e0005258. PubMed

2. Naveca FG, Nascimento VAD, Souza VC, Nunes BTD, Rodrigues DSG, Vasconcelos PFDC. Multiplexed reverse transcription real-time polymerase chain reaction for simultaneous detection of Mayaro, Oropouche, and Oropouche-like viruses. Mem Inst Oswaldo Cruz. 2017;112:510–3. PubMed

3. Lanciotti RS, Kerst AJ. Nucleic acid sequence-based amplification assays for rapid detection of West Nile and St. Louis encephalitis viruses. J Clin Microbiol. 2001;39:4506–13. PubMed

4. Lambert AJ, Martin DA, Lanciotti RS. Detection of North American eastern and western equine encephalitis viruses by nucleic acid amplification assays. J Clin Microbiol. 2003;41:379–85. PubMed
